# 2,3,5,6-Tetra­fluoro-1,4-bis­(2-pyridylmethyl­eneamino­meth­yl)benzene

**DOI:** 10.1107/S1600536809001172

**Published:** 2009-01-17

**Authors:** Ming-Yang He, Chao Li, Xu-Jie Yang, Lu-De Lu, Xin Wang

**Affiliations:** a School of Chemistry and Chemical Engineering, Nanjing University of Science and Technology, Nanjing 210093, People’s Republic of China; b Key Laboratory of Fine Petro-chemical Technology, Jiangsu Polytechnic University, Changzhou 213164, People’s Republic of China

## Abstract

The title compound, C_20_H_14_F_4_N_4_, is a flexible bis-pyridine-type ligand with an extended fluorinated spacer group between the two pyridyl functions. The centroid of the central aromatic ring is situated on a crystallographic center of inversion. The dihedral angle between the pyridine ring and the central benzene ring is 63.85 (9)°. The crystal structure exhibits inter­molecular C—H⋯F hydrogen-bonding inter­actions.

## Related literature

For background information on bis-pyridine-type Schiff base ligands see: Barboiu *et al.* (2006 ▶); Keegan *et al.* (2002 ▶); Yue *et al.* (2004 ▶). Haga *et al.* (1985 ▶) describe the synthesis of the title compound.
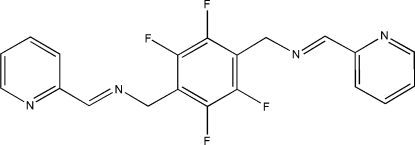


## Experimental

### 

#### Crystal data

C_20_H_14_F_4_N_4_
*M* *_r_* = 386.35
Monoclinic, 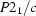

*a* = 9.637 (3) Å
*b* = 7.783 (3) Å
*c* = 12.070 (4) Å
β = 105.940 (4)°
*V* = 870.5 (5) Å^3^
*Z* = 2
Mo *K*α radiation
μ = 0.12 mm^−1^
*T* = 296 (2) K
0.26 × 0.24 × 0.22 mm

#### Data collection

Bruker SMART CCD area-detector diffractometer
Absorption correction: multi-scan (*SADABS*; Bruker, 2000 ▶) *T*_min_ = 0.963, *T*_max_ = 0.974
7194 measured reflections
2014 independent reflections
1341 reflections with *I* > 2σ(*I*)
*R*_int_ = 0.037

#### Refinement

*R*[*F* ^2^ > 2σ(*F* ^2^)] = 0.045
*wR*(*F* ^2^) = 0.151
*S* = 1.05
2014 reflections
127 parameters
H-atom parameters constrained
Δρ_max_ = 0.20 e Å^−3^
Δρ_min_ = −0.16 e Å^−3^

### 

Data collection: *SMART* (Bruker, 2000 ▶); cell refinement: *SAINT* (Bruker, 2000 ▶); data reduction: *SAINT*; program(s) used to solve structure: *SHELXTL* (Sheldrick, 2008 ▶); program(s) used to refine structure: *SHELXTL*; molecular graphics: *SHELXTL*; software used to prepare material for publication: *SHELXTL*.

## Supplementary Material

Crystal structure: contains datablocks I, global. DOI: 10.1107/S1600536809001172/im2091sup1.cif

Structure factors: contains datablocks I. DOI: 10.1107/S1600536809001172/im2091Isup2.hkl

Additional supplementary materials:  crystallographic information; 3D view; checkCIF report

## Figures and Tables

**Table 1:** Hydrogen-bond geometry (Å, °)

*D*—H⋯*A*	*D*—H	H⋯*A*	*D*⋯*A*	*D*—H⋯*A*
C2—H2⋯F2^i^	0.93	2.53	3.370 (3)	151

Symmetry code: (i) 
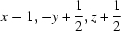
.

**Fig. 1.**
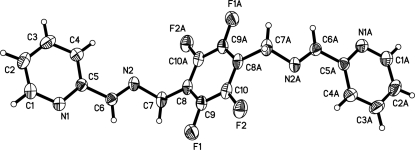
The molecular structure of the title compound showing the atom-numbering scheme. Displacement ellipsoids are drawn at the 30% probability level [symmetry code: (A) 2 - x, - y, - z].

**Fig. 2.**
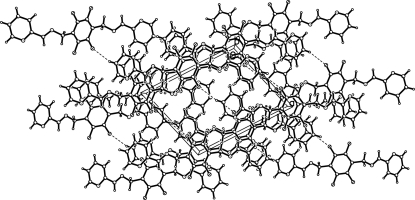
A packing diagram of the title molecule (Dashed lines indicate hydrogen bonds).

## supplementary crystallographic information

### Comment

Bis-pyridine type bidentate Schiff base ligands have been utilized intensively to assemble various coordination polymers with interesting topologies and fascinating structural diversities (Barboiu *et al.*, 2006; Keegan *et al.*, 2002; Yue *et al.*, 2004). We report here the crystal structure of the title compound, (I).

A perspective view of (I), including the atomic numbering scheme, is shown in Fig. 1. (I) crystallizes around a crystallographic center of inversion with a half molecule in the asymmetric unit. The bond lengths and angles are within normal ranges. The terminal pyridyl groups are coplanar, and they form a dihedral angle of 63.85 (9)° with the central benzene ring.

### Experimental

The title compound was synthesized and purified according to the method described by by Haga *et al.* (1985) performing a condensation of pyridine-2-carboxaldehyde and 2,3,5,6-tetrafluoro-1,4-benzenedimethanamine (yield 83%). Colorless block single crystals (m.p. 465.1–465.3 K) suitable for X-ray analysis were obtained by slow evaporation of a methanolic solution at room temperature. Analysis calculated for C_20_H_18_F_4_N_4_: C 61.54, H 4.62, N 14.36%; found: C 62.26, H 3.64, N 14.45%. IR (KBr pellet, cm^-1^): 3445 (*b*), 3087 (*m*), 3018 (*m*), 2922 (*m*), 2868 (*m*), 1639 (*s*), 1586 (*s*), 1568 (*s*), 1489 (*s*), 1470 (*s*), 1436 (*m*), 1372 (*m*), 1335 (*m*), 1275 (*m*), 1211 (*m*), 1148 (*w*), 1063 (*s*), 1013 (*m*), 987 (*s*), 892 (*s*), 776 (*s*), 742 (*m*), 697 (*m*), 621 (*w*), 590 (*m*), 509 (*m*), 413 (*w*).

### Refinement

H atoms were assigned to calculated positions, with C—H = 0.97 (methylene) and 0.93 Å (aromatic), and refined using a riding model, with *U*_iso_(H) = 1.2 *U*_eq_(C).

### Crystal data

**Table:**

C_20_H_14_F_4_N_4_	*F*(000) = 396
*M**_r_* = 386.35	*D*_x_ = 1.474 Mg m^−^^3^
Monoclinic, *P*2_1_/*c*	Mo *K*α radiation, λ = 0.71073 Å
Hall symbol: -P 2ybc	Cell parameters from 3894 reflections
*a* = 9.637 (3) Å	θ = 2.6–27.2°
*b* = 7.783 (3) Å	µ = 0.12 mm^−^^1^
*c* = 12.070 (4) Å	*T* = 296 K
β = 105.940 (4)°	Block, colorless
*V* = 870.5 (5) Å^3^	0.26 × 0.24 × 0.22 mm
*Z* = 2	

### Data collection

**Table:**

Bruker SMART CCD area-detector diffractometer	2014 independent reflections
Radiation source: fine-focus sealed tube	1341 reflections with *I* > 2σ(*I*)
graphite	*R*_int_ = 0.037
φ and ω scans	θ_max_ = 27.6°, θ_min_ = 2.2°
Absorption correction: multi-scan (*SADABS*; Bruker, 2000)	*h* = −12→12
*T*_min_ = 0.963, *T*_max_ = 0.974	*k* = −10→10
7194 measured reflections	*l* = −15→15

### Refinement

**Table:**

Refinement on *F*^2^	Primary atom site location: structure-invariant direct methods
Least-squares matrix: full	Secondary atom site location: difference Fourier map
*R*[*F*^2^ > 2σ(*F*^2^)] = 0.045	Hydrogen site location: inferred from neighbouring sites
*wR*(*F*^2^) = 0.151	H-atom parameters constrained
*S* = 1.05	*w* = 1/[σ^2^(*F*_o_^2^) + (0.0739*P*)^2^ + 0.1642*P*] where *P* = (*F*_o_^2^ + 2*F*_c_^2^)/3
2014 reflections	(Δ/σ)_max_ < 0.001
127 parameters	Δρ_max_ = 0.20 e Å^−^^3^
0 restraints	Δρ_min_ = −0.16 e Å^−^^3^

### Special details

**Table:**

Geometry. All e.s.d.'s (except the e.s.d. in the dihedral angle between two l.s. planes) are estimated using the full covariance matrix. The cell e.s.d.'s are taken into account individually in the estimation of e.s.d.'s in distances, angles and torsion angles; correlations between e.s.d.'s in cell parameters are only used when they are defined by crystal symmetry. An approximate (isotropic) treatment of cell e.s.d.'s is used for estimating e.s.d.'s involving l.s. planes.
Refinement. Refinement of *F*^2^ against ALL reflections. The weighted *R*-factor *wR* and goodness of fit *S* are based on *F*^2^, conventional *R*-factors *R* are based on *F*, with *F* set to zero for negative *F*^2^. The threshold expression of *F*^2^ > σ(*F*^2^) is used only for calculating *R*-factors(gt) *etc*. and is not relevant to the choice of reflections for refinement. *R*-factors based on *F*^2^ are statistically about twice as large as those based on *F*, and *R*- factors based on ALL data will be even larger.

### Fractional atomic coordinates and isotropic or equivalent isotropic displacement parameters (Å^2^)

**Table:**

	*x*	*y*	*z*	*U*_iso_*/*U*_eq_	
C1	0.6996 (3)	0.1926 (3)	0.52870 (19)	0.0733 (6)	
H1	0.7326	0.2286	0.6049	0.088*	
C2	0.5633 (3)	0.2358 (3)	0.4693 (2)	0.0781 (7)	
H2	0.5047	0.2981	0.5044	0.094*	
C3	0.5132 (2)	0.1855 (3)	0.3558 (2)	0.0761 (7)	
H3	0.4201	0.2127	0.3128	0.091*	
C4	0.6041 (2)	0.0941 (3)	0.30767 (18)	0.0624 (5)	
H4	0.5734	0.0591	0.2311	0.075*	
C5	0.74130 (19)	0.0548 (2)	0.37431 (15)	0.0482 (4)	
C6	0.8435 (2)	−0.0426 (2)	0.32794 (16)	0.0507 (4)	
H6	0.9325	−0.0738	0.3770	0.061*	
C7	0.9207 (2)	−0.1847 (3)	0.18519 (18)	0.0643 (6)	
H7A	0.8812	−0.2964	0.1577	0.077*	
H7B	1.0053	−0.2024	0.2496	0.077*	
C8	0.9627 (2)	−0.0904 (2)	0.08987 (16)	0.0538 (5)	
C9	1.0951 (2)	−0.0132 (3)	0.10554 (16)	0.0547 (5)	
C10	1.1317 (2)	0.0740 (2)	0.01844 (18)	0.0556 (5)	
F1	1.19419 (14)	−0.02363 (18)	0.20914 (11)	0.0756 (4)	
F2	1.26344 (13)	0.14574 (17)	0.04184 (11)	0.0762 (4)	
N1	0.79039 (18)	0.1014 (2)	0.48488 (14)	0.0622 (5)	
N2	0.81318 (17)	−0.0848 (2)	0.22290 (14)	0.0567 (4)	

### Atomic displacement parameters (Å^2^)

**Table:**

	*U*^11^	*U*^22^	*U*^33^	*U*^12^	*U*^13^	*U*^23^
C1	0.0789 (15)	0.0860 (16)	0.0611 (12)	0.0097 (13)	0.0293 (11)	−0.0086 (11)
C2	0.0766 (15)	0.0762 (16)	0.0951 (18)	0.0164 (12)	0.0465 (13)	−0.0028 (13)
C3	0.0537 (11)	0.0797 (16)	0.0951 (18)	0.0162 (11)	0.0207 (11)	0.0089 (13)
C4	0.0584 (11)	0.0667 (13)	0.0620 (12)	0.0040 (10)	0.0161 (9)	−0.0002 (10)
C5	0.0519 (10)	0.0463 (10)	0.0503 (10)	−0.0013 (8)	0.0206 (8)	0.0049 (8)
C6	0.0498 (10)	0.0523 (11)	0.0539 (10)	−0.0006 (8)	0.0207 (8)	0.0059 (8)
C7	0.0798 (13)	0.0555 (12)	0.0732 (13)	0.0025 (10)	0.0472 (11)	0.0011 (10)
C8	0.0672 (12)	0.0464 (11)	0.0597 (11)	−0.0007 (9)	0.0373 (10)	−0.0084 (8)
C9	0.0618 (11)	0.0549 (11)	0.0537 (10)	−0.0018 (9)	0.0267 (9)	−0.0124 (8)
C10	0.0577 (11)	0.0512 (11)	0.0688 (12)	−0.0102 (9)	0.0354 (10)	−0.0153 (9)
F1	0.0769 (8)	0.0893 (10)	0.0619 (8)	−0.0030 (7)	0.0213 (6)	−0.0061 (6)
F2	0.0663 (7)	0.0819 (9)	0.0898 (9)	−0.0222 (6)	0.0376 (7)	−0.0141 (7)
N1	0.0599 (10)	0.0747 (11)	0.0535 (9)	0.0079 (8)	0.0179 (7)	−0.0038 (8)
N2	0.0602 (9)	0.0624 (10)	0.0566 (9)	−0.0033 (8)	0.0316 (7)	0.0011 (7)

### Geometric parameters (Å, °)

**Table:**

C1—N1	1.343 (3)	C6—H6	0.9300
C1—C2	1.355 (3)	C7—N2	1.464 (2)
C1—H1	0.9300	C7—C8	1.511 (3)
C2—C3	1.378 (3)	C7—H7A	0.9700
C2—H2	0.9300	C7—H7B	0.9700
C3—C4	1.375 (3)	C8—C9	1.375 (3)
C3—H3	0.9300	C8—C10^i^	1.379 (3)
C4—C5	1.380 (3)	C9—F1	1.351 (2)
C4—H4	0.9300	C9—C10	1.376 (3)
C5—N1	1.338 (2)	C10—F2	1.344 (2)
C5—C6	1.470 (3)	C10—C8^i^	1.379 (3)
C6—N2	1.264 (2)		
			
N1—C1—C2	124.4 (2)	N2—C7—C8	109.82 (16)
N1—C1—H1	117.8	N2—C7—H7A	109.7
C2—C1—H1	117.8	C8—C7—H7A	109.7
C1—C2—C3	118.6 (2)	N2—C7—H7B	109.7
C1—C2—H2	120.7	C8—C7—H7B	109.7
C3—C2—H2	120.7	H7A—C7—H7B	108.2
C4—C3—C2	118.5 (2)	C9—C8—C10^i^	115.87 (17)
C4—C3—H3	120.7	C9—C8—C7	122.69 (19)
C2—C3—H3	120.7	C10^i^—C8—C7	121.43 (18)
C3—C4—C5	119.2 (2)	F1—C9—C8	119.61 (17)
C3—C4—H4	120.4	F1—C9—C10	118.19 (18)
C5—C4—H4	120.4	C8—C9—C10	122.21 (19)
N1—C5—C4	122.76 (18)	F2—C10—C9	118.03 (19)
N1—C5—C6	115.41 (16)	F2—C10—C8^i^	120.04 (17)
C4—C5—C6	121.82 (18)	C9—C10—C8^i^	121.93 (18)
N2—C6—C5	121.41 (17)	C5—N1—C1	116.50 (17)
N2—C6—H6	119.3	C6—N2—C7	117.46 (17)
C5—C6—H6	119.3		
			
N1—C1—C2—C3	−0.8 (4)	C10^i^—C8—C9—C10	−0.5 (3)
C1—C2—C3—C4	−0.2 (4)	C7—C8—C9—C10	−179.29 (17)
C2—C3—C4—C5	0.5 (4)	F1—C9—C10—F2	−0.8 (3)
C3—C4—C5—N1	0.3 (3)	C8—C9—C10—F2	179.80 (16)
C3—C4—C5—C6	179.96 (19)	F1—C9—C10—C8^i^	179.86 (16)
N1—C5—C6—N2	−175.63 (17)	C8—C9—C10—C8^i^	0.5 (3)
C4—C5—C6—N2	4.7 (3)	C4—C5—N1—C1	−1.2 (3)
N2—C7—C8—C9	108.5 (2)	C6—C5—N1—C1	179.09 (18)
N2—C7—C8—C10^i^	−70.3 (2)	C2—C1—N1—C5	1.5 (4)
C10^i^—C8—C9—F1	−179.83 (16)	C5—C6—N2—C7	−178.80 (16)
C7—C8—C9—F1	1.3 (3)	C8—C7—N2—C6	−122.2 (2)

Symmetry codes: (i) −*x*+2, −*y*, −*z*.

### Hydrogen-bond geometry (Å, °)

**Table:**

*D*—H···*A*	*D*—H	H···*A*	*D*···*A*	*D*—H···*A*
C2—H2···F2^ii^	0.93	2.53	3.370 (3)	151

Symmetry codes: (ii) *x*−1, −*y*+1/2, *z*+1/2.
